# Breastfeeding patterns and total volume of human milk consumed influence the development of the infant oral microbiome

**DOI:** 10.1080/20002297.2025.2469892

**Published:** 2025-02-25

**Authors:** Roaa A. Arishi, Zoya Gridneva, Sharon L. Perrella, Ali S. Cheema, Ching T. Lai, Matthew S. Payne, Donna T. Geddes, Lisa F. Stinson

**Affiliations:** a School of Molecular Sciences, The University of Western Australia, Crawley, WA, Australia; b School of Molecular Sciences, ABREAST Network, Perth, WA, Australia; c School of Molecular Sciences, UWA Centre for Human Lactation Research and Translation, Crawley, WA, Australia; d Ministry of Education, Riyadh, Saudi Arabia; e The Kids Research Institute Australia, Nedlands, WA, Australia; f Division of Obstetrics and Gynaecology, The University of Western Australia, Crawley, WA, Australia

**Keywords:** Oral microbiome, breastfeeding, human milk intake, breastfeeding practices, infant microbiome

## Abstract

**Background:**

The oral microbiome of breastfed infants is distinct from that of formula-fed infants. However, breastfeeding characteristics, such as time spent breastfeeding (min/24 h), breastfeeding frequency (number of breastfeeds per day), and human milk intake (ml/day) vary significantly between breastfeeding dyads.

**Objectives:**

Given that human milk and breastfeeding exposures likely influence early colonisation of the infant oral microbiome, this study aimed to elucidate the impact of breastfeeding characteristics on the development of the infant oral microbiome.

**Materials and methods:**

Oral swabs (*n* = 55) were collected from infants at three months of age, alongside breastfeeding data collected over a 24-hour period. Bacterial DNA profiles were analysed using full-length 16S rRNA gene sequencing.

**Results:**

Variations in breastfeeding characteristics contributed to differences in microbial community structure. Total breastfeeding duration (min/24 h) was positively associated with Bifidobacterium longum and Lactobacillus gasseri, while breastfeeding frequency was negatively associated with Veillonella sp. Additionally, human milk intake (ml/24 h) was negatively associated with Streptococcus parasanguinis.

**Conclusion:**

These findings underscore the significant influence of early life feeding practices on oral microbial communities and emphasise the importance role of breastfeeding in shaping the oral microbiome during early life.

## Key message

Breastfeeding characteristics and volume of human milk consumed influence early development of the infant oral microbiome.

## Introduction

The oral cavity microbiome has been found to be influenced by various factors, including delivery mode [1–5], nutrition [6–8], and intrapartum antibiotic prophylaxis [9,10]. Human milk is compositionally complex, consisting of nutritional elements, immune components, and microbial communities [11–13]. As a primary source of microbial exposure for breastfed infants, human milk contributes to the establishment of the infant oral microbiome [14]. Indeed, several taxa characteristic of the infant oral microbiome are highly abundant in human milk, including *Streptococcus mitis*, *Streptococcus salivarius*, *Gemella* spp., and *Rothia mucilaginosa* [14]. While it is not clear whether these human milk bacteria originate from the oral cavity or from other sources [15–23], there is evidence of bi-directional transfer of various species between human milk and the infant oral microbiome [24,25]. As such, studies have shown that the frequency of direct breastfeeding (feeding directly from the breast) is associated with microbial communities in human milk [26,27]. Increased frequency of direct breastfeeding has been associated with human milk beta diversity and a decreased relative abundance of *Corynebacterium* and *Staphylococcus* (*n* = 93 non-exclusively breastfed infants) [27]. Another study showed that an increased frequency of breastfeeding and physical contact with other caregivers was associated with decreased richness of human milk in 51 exclusively breastfed infants of ages 3 weeks to 5.5 months [26]. However, to date, no studies have explored the effects of breastfeeding characteristics on the infant oral microbiome. Therefore, the aim of this study was to examine associations of breastfeeding frequency, total 24 h breastfeeding duration, and 24 h human milk intake with the infant oral microbiome.

## Material and methods

### Study design

Pregnant women were enrolled to participate in the BLOSOM cohort (Breastfeeding Longitudinal Observational Study of Mothers and kids), as previously described [28]. Participants provided informed written consent. The study was approved by the Human Research Ethics Committee at The University of Western Australia (RA/4/20/4023). For this sub-study, we analysed infant oral microbiome data and breastfeeding characteristics of 55 mother-infant pairs collected at the 3-month time point.

### Sample and data collection

At enrolment, mothers completed a background questionnaire that include their demographics and health history. Within the first week after birth early feeding data was collected and feeding practices were collected monthly thereafter up to 6 months after birth.

Mothers received written instructions for collecting their infant’s oral samples as previously described [29]. Oral swabs were collected a minimum of one hour after infant feeding. The samples were obtained using COPAN E-swabs, which were firmly rubbed up and down and in a circular motion against the inside of the cheek 10 times. This procedure was then repeated on the opposite cheek with the same swab. The swabs were removed from the mouth without touching other surfaces and stored in the participant’s home refrigerator for up to 18 h before being transported on ice to the laboratory, where they were immediately processed. The oral swabs were eluted into 1 ml of the collection media (Liquid Amies) by vortexing for 5 seconds, then aliquoted into sterile tubes and stored at −80°C until further analysis.

### 24-h human milk intake

Infant milk intake was measured at three months postnatal using 24-hour test weighs as previously described [30]. Briefly, mothers recorded the time and weighed their infants before and after each feed using electronic baby scales (Medela, resolution 2 g, accuracy ±0.034%). Human milk intake, in grams, was calculated by subtracting the pre-feed weight from the post-feed weight.

### 24 h breastfeeding duration and frequency

Total 24 h breastfeeding duration was calculated as the cumulative number of minutes spent breastfeeding over a 24-hour period. Breastfeeding frequency is defined as the number of breastfeeding sessions, where a session involves feeding from either one breast or both breasts, as long as the infant starts feeding from the second breast within 30 minutes of finishing with the first [31]. Participants recorded the start time and the end time of the feed and either wrote the time of the feed down or entered it into the study website (REDCap) [31].

### Infant oral microbiome analysis

Liquid Amies was vortexed for 5 seconds to release cells from the swab. The swab media (Liquid Amies) was then centrifuged at 40,000 × g for 5 minutes at 4°C, and the resulting supernatant was carefully removed. DNA was extracted from the cell pellet using the QIAGEN MagAttract Microbial DNA Isolation Kit. This method includes a bead beating step to enhance DNA yield. A negative extraction control (reagents only) was included in each batch.

Full-length amplification of the 16S rRNA gene was performed using asymmetrically barcoded primers 27F and 1492 R. The PCR reactions, each 30 µL, contained 0.75 µL each of DTT and dsDNase (from the ArcticZymes PCR decontamination kit), 0.3 μM of both forward and reverse barcoded primers, 1X AccuStart II ToughMix, 3 µL of nuclease-free water, and 6 µL of template. All master mix reagents were treated with the ArcticZymes PCR Decontamination kit before adding the template. Each batch included a no-template control. The PCR cycling conditions comprised an initial heating step at 94°C for 3 minutes, followed by 35 cycles of 94°C for 30 seconds, 52°C for 30 seconds, and 72°C for 2 minutes, with a final extension at 72°C for 5 minutes. To verify the presence and size of amplicons, PCR products were visualised using the QIAxcel capillary gel electrophoresis system with a DNA High-Resolution cartridge. The barcoded amplicons were normalised, pooled into multiplexed groups, and purified and concentrated with Macherey-Nagel NucleoMag NGS beads. Finally, the purified amplicon pools were sequenced using a PacBio Sequel II at the Australian Genome Research Facility (AGRF).

Sequence data were analysed using Mothur v.1.48.0 [32]. The raw sequences were filtered based on length (1336–1743 bp) and homopolymer runs (≤9). These sequences were aligned with the SILVA reference alignment v132 [33], and VSEARCH was used to remove chimeric sequences [34]. Initial classification was performed with the SILVA taxonomy database (v132) [33] at a confidence threshold of 80%, excluding non-bacterial sequences. The remaining sequences were then grouped into operational taxonomic units (OTUs). OTUs with an average relative abundance exceeding 0.5% (*n* = 15) were identified using BLAST [35], applying a 97% sequence identity and 99% sequence coverage cutoff. Sequences from the negative extraction and template controls are listed in Supplementary Table S1.

### Data analysis

Diversity analyses were conducted on subsampled data. Subsampling was carried out at 2882 reads, which provided an average coverage of 85.9% and excluded 17 low-yield samples. Alpha diversity was evaluated using OTU-level richness and Shannon diversity. Beta diversity was measured using Bray-Curtis dissimilarity. Associations between breastfeeding characteristics and the infant oral microbiome at 3 months of age were assessed using centre log ratio (CLR)-transformed OTU data. linear regression model, was used to examine the relationship between breastfeeding characteristics and the relative abundance of microbial taxa [36]. All statistical analyses were conducted using R [37].

To examine how breastfeeding characteristics relate to the oral microbiome of infants at 3 months of age, we used linear regression model with the lm function in R. We fitted models for each of the 15 OTUs studied, as well as for Shannon diversity and richness. The explanatory variables included 24-hour human milk intake, breastfeeding frequency (number of breastfeeds per 24 h), and total breastfeeding duration (min/24 h). To adjust for multiple comparisons, we applied the Bonferroni correction to the p-values. The adjusted p-values are presented in Supplementary Table S2 and reported throughout the text. The association between different breastfeeding characteristics and the overall structure of the infant oral microbiome was explored using PERMANOVA with the adonis2 function from the R package vegan, employing 999 permutations [38]. Significance level was set at *p* < 0.05.

## Results

Maternal and infant characteristics are detailed in Table 1.

**Table 1.** Maternal and infant characteristics (*n* = 55).

Characteristic	Mean (range) or n (%)
Maternal age at birth (years)	32.5 (25–46)
Maternal ethnicity Caucasian Other	49 (89.1%) 6 (10.9%)
Birth mode Vaginal Elective caesarean Non-elective caesarean	41 (75%) 8 (14%) 6 (11%)
Intrapartum antibiotic exposure Yes	22 (40%)
Maternal pre-pregnancy BMI (kg/m^2^)	24.0 (15.6–40.1)
Birth season Spring Summer Autumn Winter	15 (27.3%) 2 (3.6%) 22 (40.0%) 16 (29.1%)
Infant sex Female	31 (56.4%)
Birth weight (grams)	3521.2 (2540–4357)
Birth length (cm)	50.9 (46–58)
24-h milk intake at 3 months (grams)	759.6 (348–1344)
Feeding mode at 3 months Fully breastfed Predominantly breastfed (mostly human milk, some formula)	53 (96.4%) 2 (3.6%)
Pacifier use within the first postnatal week Yes	10 (18.2%)
Siblings of infant in the home Yes	44 (80%)
Furry pets in the home Yes	33 (60%)

### The composition of the oral microbiome at 3 months of age

**Figure 1.**
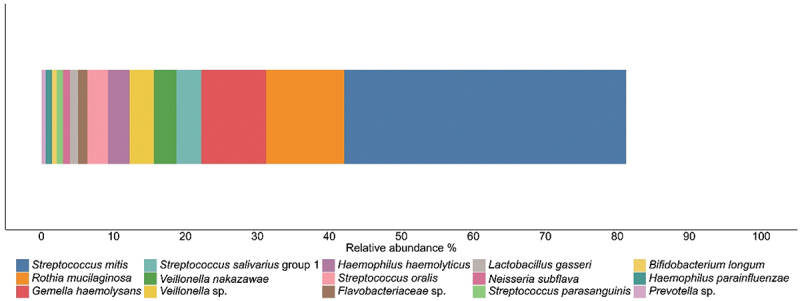
Composition of the breastfed infant oral microbiome at 3 months of age. OTUs with an average relative abundance of > 0.5% are displayed.

At three months of age, *Streptococcus* was the most dominant bacterial genus, making up 46.2% of the infant oral microbiome profiles. At the OTU level, *S. mitis* (39.1%), *R. mucilaginosa* (10.9%), and *Gemella haemolysans* (8.99%) were identified as the most abundant taxa (Figure 1). Exclusive breastfeeding was the predominant feeding mode in this population, with 53 infants (96.4%) fully breastfed. Among these, only four infants received expressed breast milk via a bottle for a single feeding within the 24-hour period. In contrast, only 2 infants (3.6%) received mixed feeding, with small volumes of formula supplementation (130 mL and 150 mL) consumed over a 24-hour period.

**Figure 2.**
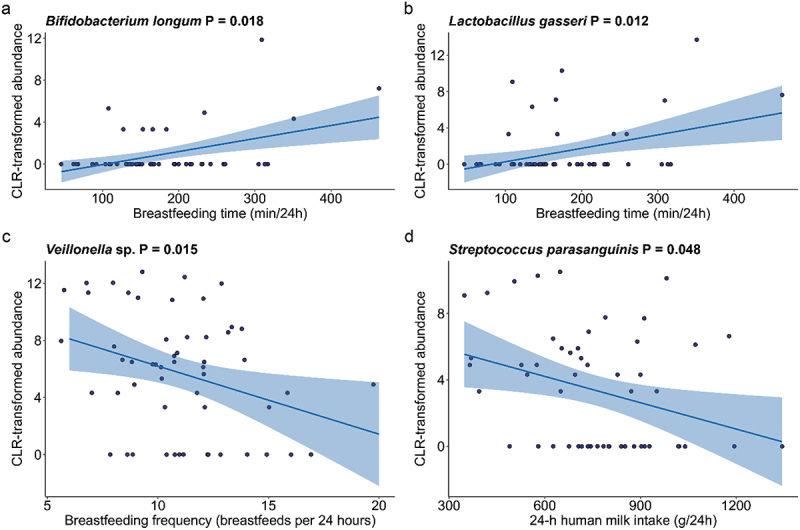
Associations between breastfeeding characteristics and the infant oral microbiome at 3 months of age. (a, b) OTUs associated with breastfeeding duration. (c) OTU associated with breastfeeding frequency. (d) OTU associated with human milk intake. All p-values are Bonferroni-adjusted for multiple comparisons.

**Figure 3.**
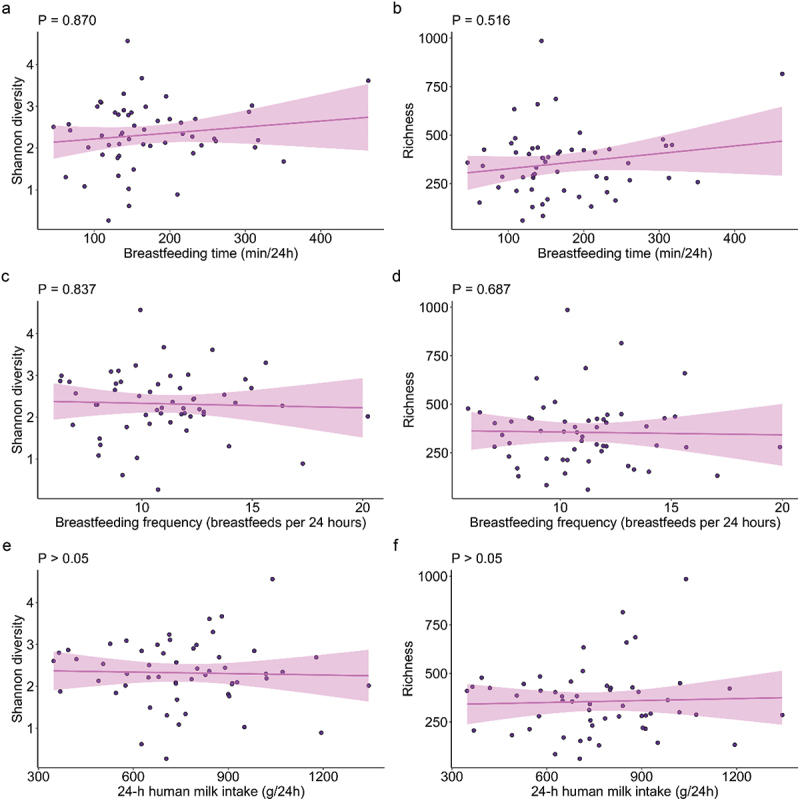
No significant associations were observed between alpha diversity metrics and breastfeeding characteristics. (a) Shannon diversity and (b) richness in relation to breastfeeding duration. (c) Shannon diversity and (d) richness in relation to breastfeeding frequency. (e) Shannon diversity and (f) richness in relation to 24-hour human milk intake. All p-values are Bonferroni-adjusted for multiple comparisons.

**Figure 4.**
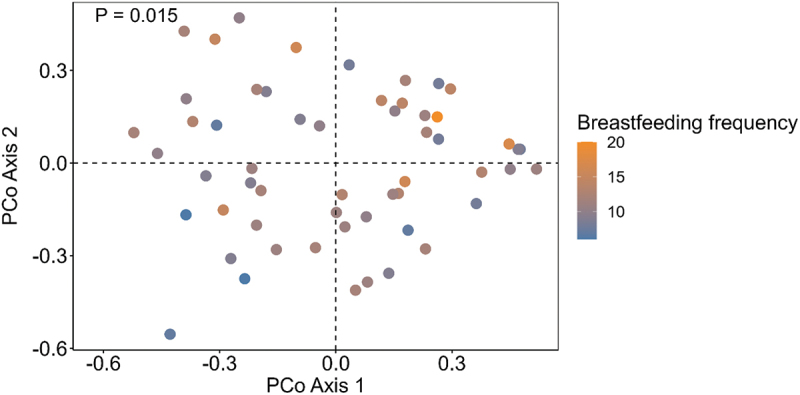
PCoA plot of the Bray-Curtis distances for infant oral samples at 3 months of age, based on breastfeeding frequency. The colour gradient indicates number of breastfeeding sessions per 24 h.

Significant associations were identified between breastfeeding characteristics and the infant oral microbiome (Supplementary Table S2). The total duration that the infant spent breastfeeding (min/24 h) was positively associated with the abundances of *Bifidobacterium longum* (*p* = 0.018) and *Lactobacillus gasseri* (*p* = 0.012) (Figure 2a and b). Breastfeeding frequency was negatively associated with *Veillonella* sp. (*p* = 0.015) (Figure 2c). Infant milk intake was negatively associated with the abundance of *Streptococcus parasanguinis* (*p* = 0.048) (Figure 2d). Alpha diversity metrics (Shannon diversity and richness) showed no significant differences based on breastfeeding characteristics (all *p* > 0.05) (Figure 3). Breastfeeding frequency explained a significant amount of variance within the infant oral microbiome (PERMANOVA R^2^ = 0.042, *p* = 0.01) (Figure 4, Supplementary Table S3).

## Discussion

The oral cavity of a breastfed infant is consistently exposed to maternal bacteria predominantly via breastfeeding [39]. The human milk microbiome also includes taxa typical of the infant oral microbiome, suggesting that breastfeeding shapes the oral microbiome through direct bacterial transfer from milk or potentially vice versa with a constant bidirectional transfer of microbes [14]. However breastfeeding characteristics, such as the volume of milk consumed, vary widely between dyads [30] and could influence oral colonisation. Within our cohort mothers spent 46 to 463 minutes breastfeeding in a 24-hour period, with longer durations associated with higher abundances of *B. longum* and *L. gasseri*. These relationships may be attributed to prolonged exposure to the bacteria present in human milk, as well as exposure to human milk oligosaccharides (HMOs); however, our data is not able to determine directionality of the association. These prebiotics have a bifidogenic effect, promoting the growth of *Bifidobacterium* while inhibiting pathogenic bacteria [40,41]. While various *Bifidobacterium* species are able to utilise HMOs as a sole carbon source, *L. gasseri* does not ferment HMOs [42]; however, it is able to take advantage of other structures derived from HMO degradation [43]. Extended breastfeeding durations may therefore increase the exposure to HMOs within the oral cavity, providing a more favourable environment for these bacteria to thrive.

The number of breastfeeding sessions within 24 h varied widely between the dyads in our study [6–20]. Breastfeeding frequency may also play a role in shaping the infant oral microbiome. Indeed, we found that breastfeeding frequency explained significant variance in the infant oral microbiome, with differences in beta diversity and the abundance of *Veillonella* sp. Whilst there are no other published data regarding relationships of breastfeeding frequency with the infant oral microbiome, two studies have explored the impact of breastfeeding frequency on the human milk microbiome. In non-exclusively breastfed infants, the frequency of direct breastfeeding was associated with the beta diversity of the human milk microbiome and a decreased relative abundance of *Corynebacterium* and *Staphylococcus* [27]. In exclusively breastfed infants (*n* = 46) aged 3 to 24 weeks postpartum, increased breastfeeding frequency was associated with decreased richness of the human milk microbiome, as well as differential associations with the abundances of *Bifidobacterium*, *Micrococcus*, *Pedobacter*, *Acidocella*, and *Achromobacter* [26]. Whilst the effect of breastfeeding frequency on the human milk microbiome may be related to the transfer of microbes to the breast from the oral cavity, the oral cavity itself is likely also influenced by increased clearance of microbes and/or exposure to antibacterial components in human milk. Future studies analysing both the human milk microbiome and infant oral microbiome may aid in clarifying the impacts of human milk feeding habits on the colonisation of the infant oral cavity.

Although rarely measured in other studies, we found that the volume of human milk consumed by the infant showed a negative relationship with the abundance of *S. parasanguinis. S. parasanguinis* is generally regarded as an oral commensal that has been associated with dental plaque in adults [44]. Our results suggests that greater human milk intake volumes may inhibit the proliferation of certain microbial species, potentially due to the presence of antimicrobial components in human milk [45] or the competitive exclusion by other beneficial bacteria introduced through higher milk volume consumption [46]. Reduction of *S. parasanguinis* by human milk during tooth eruption in early life may be beneficial for the reduction of caries [47]. Breastfeeding duration of up to 17 months has also been shown to reduce the prevalence of childhood caries [48]. Further, *S. parasanguinis* has also been identified as the dominant microbe in a proportion of young children with dental caries [47]. This underscores the importance of human milk in shaping the infant oral bacterial profile, potentially in a dose-dependent manner. However, given that human milk composition varies between individuals, further investigation is needed to elucidate how variations in milk intake and composition affect the development and stability of the infant oral microbiome.

While comparisons between breastfed and formula-fed infants are commonly made in the literature, our study intentionally focused on the variability of breastfeeding characteristics within exclusively and predominantly breastfed infants, as breastfeeding behaviours vary widely even within exclusively breastfeeding dyads. This approach allows for a clearer analysis of how breastfeeding behaviours influence the oral microbiome, as the absence of formula consumption mitigates the confounding effects associated with formula feeding.

## Strengths and limitations

The strengths of this study lie in the measures of breastfeeding characteristics and human milk intake as well as the use of full-length 16S rRNA gene sequencing providing enhanced taxonomic resolution and greater accuracy in microbial identification compared to short-read sequencing methods [49,50]. The main limitation is the relatively small and ethnically homogenous cohort which restricts generalisability of our findings to the broader population. Additionally, the classification of feeding mode was based on self-reports from mothers, which may be subject to inaccuracies in reporting, particularly for night feedings. However, intention to exclusively breastfeed and to have a duration of breastfeeding of at least 12 months were recruitment criteria for this cohort. Another potential limitation is the lack of data on maternal diet, which may influence the composition of human milk and, consequently, the infant oral microbiome. Test weighing, while widely used to estimate milk intake, has limitations such as potential inaccuracies due to measurement errors or incomplete recording, which should be considered. Further, the cross-sectional design of our study limits our ability to determine causality. Although it should be noted that the 3 month timepoint has been shown to be representative of breastfeeding characteristics and milk intake from 1–6 months [31]. While our results provided initial hypothesis-forming data, future longitudinal research involving larger and more diverse populations, including mixed-fed and exclusively formula-fed infants, is needed to better understand the colonisation of the infant oral microbiome.

## Conclusion

The results of this small, hypothesis forming study suggests breastfeeding characteristics and volume of human milk consumed impact the infant oral microbiome. Further research is essential to understand the synergistic effects of human milk biochemical and microbial composition on development of the infant oral microbiome.

## Supplementary Material

Arishi et al_revised SI.docx

## Data Availability

The raw sequencing data are available in the NCBI Sequence Read Archive (SRA) under BioProject ID [PRJNA1186108].
