# Supplementary material for: Breastfeeding patterns and total volume of human milk consumed influence the development of the infant oral microbiome
Source: J Oral Microbiol. 2025 Feb 25;17(1):2469892. doi: 10.1080/20002297.2025.2469892 (PMC11864009; doi:10.1080/20002297.2025.2469892)
Supplement: Arishi et al_revised SI.docx [file ZJOM_A_2469892_SM2085.docx]

**Supplementary Table 1**: Genera detected in negative extraction controls (EC, n = 8) and negative PCR controls (NTC, n = 8). Data are read numbers.

| Genus | **EC1** | **EC2** | **EC3** | **EC4** | **EC5** | **EC6** | **EC7** | **EC8** | **NTC1** | **NTC2** | **NTC3** | **NTC4** | **NTC5** | **NTC6** | **NTC7** | **NTC8** |
| --- | --- | --- | --- | --- | --- | --- | --- | --- | --- | --- | --- | --- | --- | --- | --- | --- |
| *Actinomyces* | 2 | 2 | 0 | 0 | 3 | 1 | 1 | 0 | 1 | 1 | 0 | 0 | 1 | 1 | 0 | 0 |
| *Atopobium* | 2 | 1 | 0 | 1 | 0 | 0 | 0 | 0 | 0 | 1 | 2 | 0 | 0 | 0 | 0 | 0 |
| *Bergeyella* | 7 | 1 | 5 | 0 | 1 | 2 | 1 | 1 | 0 | 1 | 8 | 1 | 2 | 1 | 4 | 0 |
| *Gemella* | 37 | 0 | 1 | 0 | 0 | 0 | 1 | 1 | 1 | 0 | 1 | 2 | 2 | 0 | 3 | 1 |
| *Granulicatella* | 1 | 3 | 0 | 0 | 2 | 2 | 1 | 5 | 1 | 1 | 1 | 1 | 2 | 2 | 1 | 2 |
| *Haemophilus* | 3 | 0 | 0 | 0 | 0 | 0 | 0 | 0 | 0 | 0 | 0 | 0 | 0 | 0 | 0 | 0 |
| *Lactobacillus* | 1 | 1 | 1 | 0 | 1 | 1 | 0 | 0 | 0 | 1 | 3 | 0 | 1 | 1 | 0 | 1 |
| *Neisseria* | 8 | 2 | 5 | 2 | 5 | 4 | 1 | 2 | 0 | 4 | 2 | 1 | 9 | 2 | 2 | 3 |
| *Porphyromonas* | 9 | 0 | 0 | 0 | 0 | 0 | 0 | 0 | 0 | 1 | 1 | 0 | 2 | 1 | 0 | 0 |
| *Rothia* | 70 | 3 | 2 | 1 | 1 | 4 | 1 | 1 | 3 | 1 | 2 | 0 | 3 | 1 | 2 | 3 |
| Unclassified *Saccharimonadales* | 4 | 4 | 8 | 1 | 11 | 7 | 5 | 26 | 3 | 10 | 10 | 6 | 7 | 7 | 11 | 1 |
| *Sebaldella* | 1 | 0 | 0 | 0 | 0 | 1 | 0 | 0 | 0 | 0 | 0 | 0 | 0 | 0 | 0 | 0 |
| *Staphylococcus* | 3 | 0 | 3 | 0 | 6 | 5 | 1 | 0 | 2 | 4 | 1 | 2 | 3 | 2 | 5 | 0 |

| Genus | **EC1** | **EC2** | **EC3** | **EC4** | **EC5** | **EC6** | **EC7** | **EC8** | **NTC1** | **NTC2** | **NTC3** | **NTC4** | **NTC5** | **NTC6** | **NTC7** | **NTC8** |  |
| --- | --- | --- | --- | --- | --- | --- | --- | --- | --- | --- | --- | --- | --- | --- | --- | --- | --- |
| *Streptococcus* | 719 | 13 | 18 | 7 | 27 | 16 | 11 | 60 | 6 | 11 | 21 | 16 | 21 | 15 | 13 | 4 |  |
| Unclassified *Actinobacteria* | 2 | 2 | 0 | 0 | 0 | 3 | 1 | 0 | 0 | 0 | 3 | 1 | 1 | 1 | 1 | 0 |  |
| Unclassified *Bacilli* | 3 | 1 | 1 | 0 | 2 | 3 | 2 | 0 | 0 | 0 | 2 | 0 | 1 | 2 | 0 | 1 |  |
| Unclassified bacteria | 37 | 2 | 1 | 0 | 2 | 5 | 3 | 0 | 1 | 6 | 4 | 2 | 6 | 1 | 2 | 1 |  |
| Unclassified *Carnobacteriaceae* | 3 | 1 | 2 | 1 | 2 | 8 | 1 | 0 | 4 | 4 | 7 | 1 | 4 | 5 | 3 | 2 |  |
| Unclassified *Lactobacillales* | 7 | 4 | 2 | 1 | 12 | 18 | 6 | 0 | 0 | 1 | 3 | 4 | 9 | 8 | 11 | 3 |  |
| Unclassified *Leptotrichiaceae* | 1 | 1 | 0 | 0 | 3 | 0 | 0 | 3 | 1 | 0 | 2 | 0 | 2 | 0 | 1 | 0 |  |
| Unclassified *Micrococcaceae* | 6 | 0 | 0 | 0 | 0 | 0 | 0 | 0 | 0 | 0 | 2 | 0 | 0 | 0 | 0 | 0 |  |
| Unclassified *Micrococcales* | 25 | 5 | 6 | 2 | 2 | 9 | 2 | 4 | 3 | 4 | 8 | 6 | 8 | 4 | 4 | 2 |  |
| Unclassified *Neisseriaceae* | 1 | 0 | 4 | 2 | 3 | 4 | 61 | 9 | 0 | 0 | 0 | 0 | 0 | 1 | 1 | 2 |  |
| Unclassified *Porphyromonadaceae* | 1 | 3 | 1 | 0 | 4 | 1 | 3 | 1 | 1 | 2 | 0 | 1 | 7 | 2 | 4 | 0 |  |
| Unclassified *Streptococcaceae* | 33 | 1 | 4 | 0 | 2 | 0 | 0 | 0 | 0 | 1 | 1 | 0 | 0 | 0 | 2 | 0 |  |
| *Veillonella* | 549 | 8 | 9 | 2 | 6 | 7 | 6 | 18 | 3 | 8 | 4 | 11 | 8 | 8 | 5 | 2 |  |
| Others* | 1 | 7 | 6 | 9 | 51 | 57 | 95 | 33 | 6 | 13 | 4 | 9 | 41 | 47 | 27 | 15 |  |

*“Others” represents genera with ≤ 26 reads in negative controls.

**Supplementary Table 2**: Outputs from linear model for associations between breastfeeding behaviour and the infant oral microbiome. Variables with significant P-values are reported.

| **Response variable** | **Explanatory variable** | **Estimate** | **Standard error** | **P-value** | **Adjusted P-value** |
| --- | --- | --- | --- | --- | --- |
| *Streptococcus mitis* (OTU000001) | Breastfeeding time | -0.004 | 0.002 | 0.127 | 0.381 |
|  | Breastfeeding frequency | 0.116 | 0.084 | 0.170 | 0.510 |
|  | Human milk intake | 0.0004 | 0.001 | 0.627 | 1.00 |
| *Gemella haemolysans* (OTU000002) | Breastfeeding time | -0.0008 | 0.005 | 0.886 | 1.00 |
|  | Breastfeeding frequency | -0.089 | 0.156 | 0.570 | 1.00 |
|  | Human milk intake | -0.002 | 0.001 | 0.168 | 0.504 |
| *Rothia mucilaginosa* (OTU000003) | Breastfeeding time | 0.0006 | 0.006 | 0.918 | 1.00 |
|  | Breastfeeding frequency | 0.224 | 0.184 | 0.228 | 0.684 |
|  | Human milk intake | -0.002 | 0.002 | 0.335 | 1.00 |
| *Streptococcus salivarius* group1 (OTU000004) | Breastfeeding time | 0.003 | 0.007 | 0.631 | 1.00 |
|  | Breastfeeding frequency | -0.032 | 0.220 | 0.885 | 1.00 |
|  | Human milk intake | -0.002 | 0.002 | 0.279 | 0.837 |
| *Veillonella* sp. (OTU000005) | Breastfeeding time | 0.011 | 0.007 | 0.139 | 0.417 |
|  | Breastfeeding frequency | -0.606 | 0.210 | **0.005** | **0.015** |
|  | Human milk intake | -0.0004 | 0.002 | 0.853 | 1.00 |
| *Neisseria subflava* (OTU000006) | Breastfeeding time | -0.0009 | 0.006 | 0.875 | 1.00 |
|  | Breastfeeding frequency | -0.228 | 0.172 | 0.192 | 0.576 |
|  | Human milk intake | 0.001 | 0.002 | 0.590 | 1.00 |
| *Streptococcus oralis* (OTU000007) | Breastfeeding time | 0.005 | 0.007 | 0.452 | 1.00 |
|  | Breastfeeding frequency | 0.100 | 0.215 | 0.644 | 1.00 |
|  | Human milk intake | 0.005 | 0.002 | 0.055 | 0.165 |
| Haemophilus haemolyticus (OTU000008) | Breastfeeding time | 0.0003 | 0.008 | 0.964 | 1.00 |
|  | Breastfeeding frequency | -0.262 | 0.240 | 0.279 | 0.837 |
|  | Human milk intake | 0.001 | 0.002 | 0.715 | 1.00 |
| *Veillonella nakazawae* (OTU000009) | Breastfeeding time | 0.006 | 0.006 | 0.337 | 1.00 |
|  | Breastfeeding frequency | 0.058 | 0.183 | 0.752 | 1.00 |
|  | Human milk intake | -0.0006 | 0.002 | 0.765 | 1.00 |
| *Haemophilus parainfluenzae* (OTU000011) | Breastfeeding time | -0.008 | 0.006 | 0.217 | 0.651 |
|  | Breastfeeding frequency | -0.028 | 0.185 | 0.876 | 1.00 |
|  | Human milk intake | -0.0006 | 0.002 | 0.787 | 1.00 |
| Flavobacteriaceae sp. (OTU000012) | Breastfeeding time | 0.010 | 0.006 | 0.127 | 0.381 |
|  | Breastfeeding frequency | -0.369 | 0.191 | 0.059 | 0.177 |
|  | Human milk intake | -0.0008 | 0.002 | 0.713 | 1.00 |
| *Streptococcus parasanguinis* (OTU000013) | Breastfeeding time | 0.003 | 0.006 | 0.647 | 1.00 |
|  | Breastfeeding frequency | 0.045 | 0.187 | 0.807 | 1.00 |
|  | Human milk intake | -0.005 | 0.002 | **0.016** | **0.048** |
| *Prevotella* sp. (OTU000014) | Breastfeeding time | -0.003 | 0.005 | 0.506 | 1.00 |
|  | Breastfeeding frequency | -0.268 | 0.152 | 0.084 | 0.252 |
|  | Human milk intake | 0.0003 | 0.001 | 0.833 | 1.00 |
| *Bifidobacterium longum* (OTU000015) | Breastfeeding time | 0.011 | 0.004 | **0.006** | **0.018** |
|  | Breastfeeding frequency | 0.053 | 0.118 | 0.653 | 1.00 |
|  | Human milk intake | -0.001 | 0.001 | 0.305 | 0.915 |
| *Lactobacillus gasseri* (OTU000016) | Breastfeeding time | 0.017 | 0.005 | **0.004** | **0.012** |
|  | Breastfeeding frequency | -0.232 | 0.161 | 0.155 | 0.465 |
|  | Human milk intake | 0.001 | 0.001 | 0.533 | 1.00 |
| Shannon | Breastfeeding time | 0.001 | 0.001 | 0.290 | 0.870 |
|  | Breastfeeding frequency | -0.044 | 0.040 | 0.279 | 0.837 |
|  | Human milk intake | -0.0002 | 0.0004 | 0.691 | 1.00 |
| Richness | Breastfeeding time | 0.446 | 0.322 | 0.172 | 0.516 |
|  | Breastfeeding frequency | -11.064 | 9.090 | 0.229 | 0.687 |
|  | Human milk intake | 0.022 | 0.110 | 0.837 | 1.00 |

**Supplementary Table 3**: Outputs from PERMANOVA assessing the beta diversity (Bray-Curtis dissimilarity) of the oral microbiome in relation to breastfeeding behaviour

| **Explanatory variable** | **Sum Of Sqs** | R² | **F** | **P-value** |
| --- | --- | --- | --- | --- |
| Breastfeeding time | 0.199 | 0.018 | 1.017 | 0.377 |
| Breastfeeding frequency | 0.473 | 0.042 | 2.414 | **0.015** |
| Human milk intake | 0.162 | 0.014 | 0.830 | 0.603 |
